# Morphine stimulates CCL2 production by human neurons

**DOI:** 10.1186/1742-2094-3-32

**Published:** 2006-12-08

**Authors:** R Bryan Rock, Shuxian Hu, Wen S Sheng, Phillip K Peterson

**Affiliations:** 1 Center for Infectious Diseases and Microbiology Translational Research and the Department of Medicine, University of Minnesota Medical School, Minneapolis, MN, USA; 2 Division of Infectious Diseases and International Medicine, Department of Medicine, University of Minnesota Medical School, McGuire Translational Research Facility, 2001 6^th ^Street SE #3-218, Minneapolis, MN 55455, USA

## Abstract

**Background:**

Substances of abuse, such as opiates, have a variety of immunomodulatory properties that may influence both neuroinflammatory and neurodegenerative disease processes. The chemokine CCL2, which plays a pivotal role in the recruitment of inflammatory cells in the nervous system, is one of only a few chemokines produced by neurons. We hypothesized that morphine may alter expression of CCL2 by human neurons.

**Methods:**

Primary neuronal cell cultures and highly purified astrocyte and microglial cell cultures were prepared from human fetal brain tissue. Cell cultures were treated with morphine, and cells were examined by RNase protection assay for mRNA. Culture supernatants were assayed by ELISA for CCL2 protein. β-funaltrexamine (β-FNA) was used to block μ-opioid receptor (MOR)s.

**Results:**

Morphine upregulated CCL2 mRNA and protein in neuronal cultures in a concentration- and time-dependent fashion, but had no effect on CCL2 production in astrocyte or microglial cell cultures. Immunocytochemical analysis also demonstrated CCL2 production in morphine-stimulated neuronal cultures. The stimulatory effect of morphine was abrogated by β-FNA, indicating an MOR-mediated mechanism.

**Conclusion:**

Morphine stimulates CCL2 production by human neurons via a MOR-related mechanism. This finding suggests another mechanism whereby opiates could affect neuroinflammatory responses.

## Background

Substances of abuse have been shown to have a number of immunomodulatory activities [1,2], and drugs such as opiates have been implicated as a cofactor in the pathogenesis of neuroinflammatory conditions such as HIV-1 encephalitis [3]. Three classes of opioid receptors (μ, κ, and δ) have been identified in neurons, and these same receptors are found in macrophages and lymphocytes, which suggest opioids serve as communication signals between neurons and cells of the immune system. There is substantial evidence that this cross-talk between the nervous and the immune systems also involves chemokines, which along with neuropeptides and neurotransmitters, appear to function as a third major system of communication within the brain [4]. Examples of connections between the opioid and chemokine signalling systems include the demonstration that a μ-opioid receptor (MOR) selective agonist increases the expression of CCL2, CCL5 and CXCL10 mRNA and protein in human peripheral blood mononuclear cells [5] and that morphine modulates chemokine gene regulation in human astrocytes [6].

The chemokine CCL2 is an inflammatory mediator which recruits monocyte/macrophage-derived cells into areas of damage within the central nervous system (CNS). CCL2 is produced by a variety of cell types including macrophages and endothelial cells, but in the CNS, release of CCL2 has been classically attributed to astrocytes and microglia [7]. The chemotactic properties of CCL2 extend to T-lymphocytes, natural killer cells, basophils, mast cells, and dendritic cells [8]. Recent studies have also shown that neurons themselves constitutively produce CCL2. Expression of CCL2 by developing human neurons was demonstrated by immunocytochemical and western blotting methods [9]. Both CCL2 mRNA and soluble CCL2 were identified in the NT2 neuronal cell line [10]. CCL2 was also shown to be released from remote neurons in a rat nerve injury model [11], from murine neurons in a compression model [12], and from murine neurons in an ischemia model [13]. Studies of CCL2 in rat neurons have revealed that constitutive neuronal expression of CCL2 is highly regionalized and in the rat model, is found in both cholinergic and dopaminergic neurons [14]. Although the cellular source has not been definitively established, CCL2 is upregulated in such neuroinflammatory processes as HIV dementia (HAD) [8,15], experimental allergic encephalomyelitis [16], and multiple sclerosis [17] and may integral to recruitment of neural progenitors to sites of neuroinflammation [18].

Based upon the established importance of CCL2 in HAD [15,19-21] and mounting evidence that opiates foster the neuropathogenesis of HIV-1 [22], the purpose of the present study was to test the hypothesis that the opioid agonist morphine can alter CCL2 expression in human neurons. We have found that morphine robustly enhanced CCL2 expression, that this effect is unique to neurons, and that it appears to involve MORs.

## Methods

### Reagents

The following reagents were purchased from the indicated sources: fetal bovine serum (FBS) (Hyclone, Logan, UT); morphine sulfate, Dulbecco's modified Eagle medium (DMEM), penicillin, streptomycin, Hanks' balanced salt solution (HBSS), trypsin, bovine serum albumin, polyoxyethylenesorbitan monolaurate (Tween 20), PBS, and paraformaldehyde, (Sigma, St. Louis, MO); neural basal medium and B-27 serum-free supplement (Invitrogen, Carlsbad, CA); anti-neuron nuclei (NeuN; a neuronal marker) and anti-microtubule-associated protein-2 (MAP2; a neuronal marker) antibodies (Chemicon, Temecula, CA); anti-CCL2 antibodies (R&D Systems); anti-glial fibrillary protein (GFAP; an astrocyte marker) antibody (Sternberger Monoclonals, Lutherville, MD); anti-CD68 (a microglial cell marker) antibody (BD Pharmingen, San Diego, CA); β-funaltrexamine (β-FNA) (Tocris, Ellisville, MO); *trans*-3,4-dichloro-*N*-methyl-*N *[2-(1-pyrolidinyl)cyclohexyl] benzeneacetamide methanesulfonate (U50, 488; a gift from Pharmacia Corp.); anti-phosphorylated-p38 MAPK antibody (Cell Signaling, Danvers, MA); and acrylamide/bis solution (Bio-Rad, Hercules, CA).

### Cell cultures

Human fetal brain tissue was obtained from women undergoing elective abortions, in accordance with informed-consent guidelines and a protocol approved by the Human Subjects Research Committee at our institution.

Highly enriched neuronal cultures were prepared as described elsewhere [23]. In brief, 15–16-week-old cortical brain tissues were dissociated and resuspended in neural basal medium containing B-27 serum-free supplement (contains antioxidants) plus penicillin (100 U/mL) and streptomycin (100 μg/mL). Dispersed cells were plated onto collagen-coated plates (5 × 10^5^, 10^6^, or 3 × 10^6 ^cells/well in 24-, 12-, or 6-well plates, respectively) or chamber slides (4 × 10^5 ^cells/well in 4-well chambers). On day 12, these brain-cell cultures contained ~70–80% neurons (stained by anti-NeuN or anti-MAP2 antibodies), 15–25% astrocytes (stained by anti-GFAP antibody), and 3–7% microglial cells (stained by anti-CD68 antibody). For highly purified neuronal cultures, on day 5, cell cultures were treated with uridine (33.6 μg/mL) and fluorodeoxyuridine (13.6 μg/mL), followed by replacement with neural basal medium with B-27 serum-free supplement (contains antioxidants) on day 6 and every 4 days thereafter. Highly purified neuronal cultures are >95% neurons, 2–3% astrocytes, and 1–2% microglial cells.

Primary human microglia and astrocyte cultures were prepared as previously described [24]. Briefly, brain tissues from 16-to-20-week-old aborted fetuses were dissociated by trypsinization (0.25%) for 30-min and plated into 75-cm^2 ^Falcon tissue culture flasks in Dulbecco's modified Eagle's medium (DMEM) containing 10% FBS, penicillin (100 U/mL), and streptomycin (100 μg/mL). Cells were incubated for 10–14 days with weekly medium changes. Microglia floating in the medium were collected, centrifuged, and reseeded onto 6-well (2.5 × 10^6 ^cells/well), 12-well (1 × 10^6 ^cells/well), or 24-well (0.5 × 10^6 ^cells/well) tissue culture plates with fresh medium. The cultures were washed 1 h after seeding to remove non-adherent cells. Microglia cultures were comprised of cells that were >99% positive for CD68 (a human macrophage marker) and <1% positive for GFAP (an astrocyte marker). To isolate astrocytes, on day 21, flasks were shaken, washed and trypsinized with 0.25% trypsin in HBSS for 30 min at 37°. After adding FBS (final concentration 10%), centrifugation, and washing, cells were seeded into new flasks with DMEM followed by medium change after 24 h. The subculture procedure was repeated four times at a weekly interval. Astrocyte cultures were comprised of cells that were >99% GFAP-positive.

### Cell viability

To assess the effect of morphine at 10^-4 ^M (used in the immunocytochemical staining experiments) on cell viability two assays were used: Cell Death Detection ELISA^PLUS ^(Roche Diagnostics, Indianapolis, IN) and MTT (3-[4,5-dimethylthiazol-2-yl]-2,5-diphenyl tetrazolium bromide mitochondrial dehydrogenase) (Sigma) assays. The Cell Death Detection ELISA^PLUS ^assay was performed according to the manufacture's instructions. MTT was added to neuronal cultures at a final concentration of 1 mg/ml, and after 4 h of incubation, the assay was stopped by adding lysis buffer (20% SDS [w/v] in 50% N, N-dimethyl formamide, pH 4.7) followed by overnight incubation. The absorbance (O.D.) measured at 600 nm reflects mitochondrial integrity.

### RNase protection assay (RPA)

To assess chemokine mRNA expression, total RNA isolated with RNeasy^® ^mini kit (Qiagen, Valencia, CA) from highly enriched neuronal cultures was used in the multiprobe RPA according to the manufacturer's protocol (BD Biosciences PharMingen, San Diego, CA).

### ELISA

To measure CCL2 in highly enriched neuronal culture supernatants, ELISA plates (96-well) were coated with corresponding mouse anti-human antibodies (1–2 μg/ml) overnight at 4°C. The plates were blocked with 1% bovine serum albumin in PBS for 1 h at 37°C. After washing with PBS containing Tween 20, culture supernatants and a series of dilutions of CCL2 as standards were added to wells for 2 h at 37°C. Following washing, detection antibody (goat anti-human CCL2 1–2 μg/ml) was added for 90 min at 37°C followed by donkey anti-goat IgG horseradish-peroxidase conjugate (1:10,000) for 45 min. A chromogen substrate K-blue (Neogen, Lexington, KY) was then added at room temperature for color development, which was stopped with 1 M H_2_SO_4_. The plate was read at 450 nm to generate standard concentration curves for CCL2 concentration extrapolation.

### Immunocytochemical staining

Highly purified neuronal cultures on 4-chamber slides were fixed with 4% paraformaldehyde followed by blocking with 10% goat serum for 20 min at room temperature. After washing with PBS, culture slides were incubated with rabbit anti-MAP2 (1:1000) and mouse anti-CCL2 (10 μg/ml) antibodies overnight at 4°C. After washing with PBS, culture slides were incubated with Fluorescein (FITC)-conjugated goat anti-rabbit IgG (5 μg/ml for MAP2 staining) and Rhodamine Red X-conjugated goat anti-mouse IgG (5 μg/ml for CCL2 staining) (Jackson ImmunoResearch, West Grove, PA) for 60 min at room temperature. After washing with PBS, mounting medium (Vector Laboratories, Burlingame, CA) and cover slip were applied for viewing under fluorescence microscopy.

### Statistical analysis

Data are expressed as mean ± SEM or mean ± SD. For comparison of means the paired Student t-test was used. For the data using the MOR antagonist β-FNA, we performed an ANOVA analysis. Since we expected that β-FNA will inhibit the effect of morphine, but not controls, we used a saturated two-factor ANOVA model in order to estimate the possible interaction. To estimate the mean inhibitory effect of β-FNA and assess its statistical significance, we compared the mean difference in morphine-exposed and control neuronal CCL2 production in the presence and absence of β-FNA. We used Levene's test to assess the ANOVA equal variance assumption and R^2 ^to measure model fit.

## Results

### Morphine stimulates CCL2 production by human neurons

**Figure 1:**
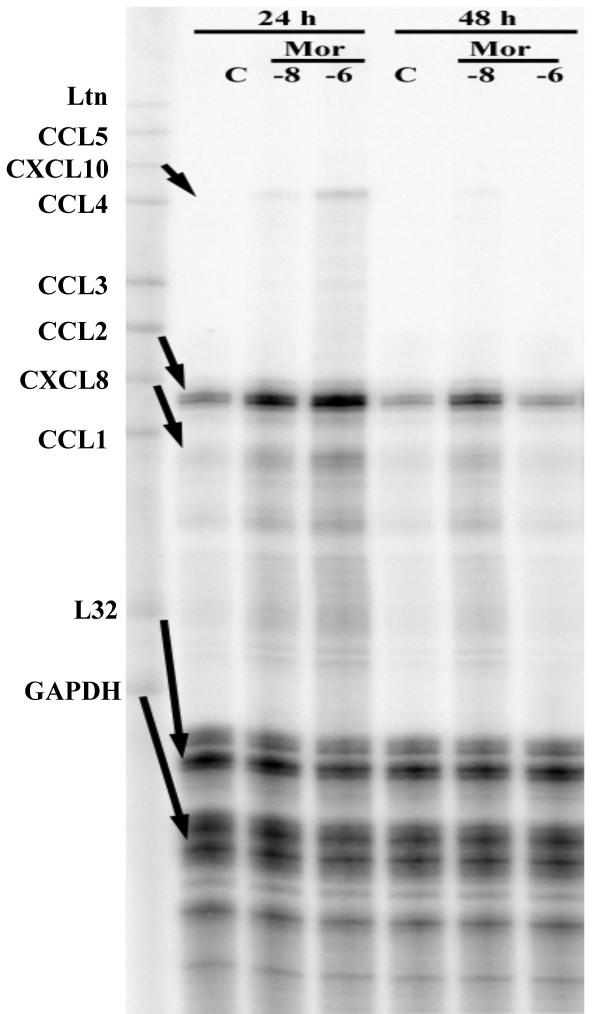
**Morphine effect on human neuronal chemokine production**. Total RNA (5 μg) isolated from control (C) and morphine (10^-8^, 10^-6 ^M at 24 and 48 h) exposed highly enriched neurons were used in RPA with a chemokine template. Ltn, lymphotactin; GAPDH glyceraldehydes 3-phosphate dehydrogenase.

**Figure 2:**
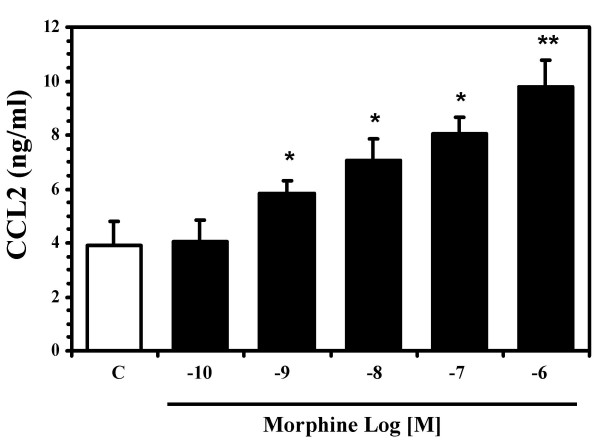
**Concentration-responses of morphine on human neuronal CCL2 production**. Cell culture supernatants were collected from highly enriched neuronal cell cultures treated with the indicated concentrations of morphine for 24 h. Data are mean ± SEM of triplicates of three separate experiments using neurons derived from different brain specimens. *P < 0.05, **P < 0.01 versus control.

**Figure 3:**
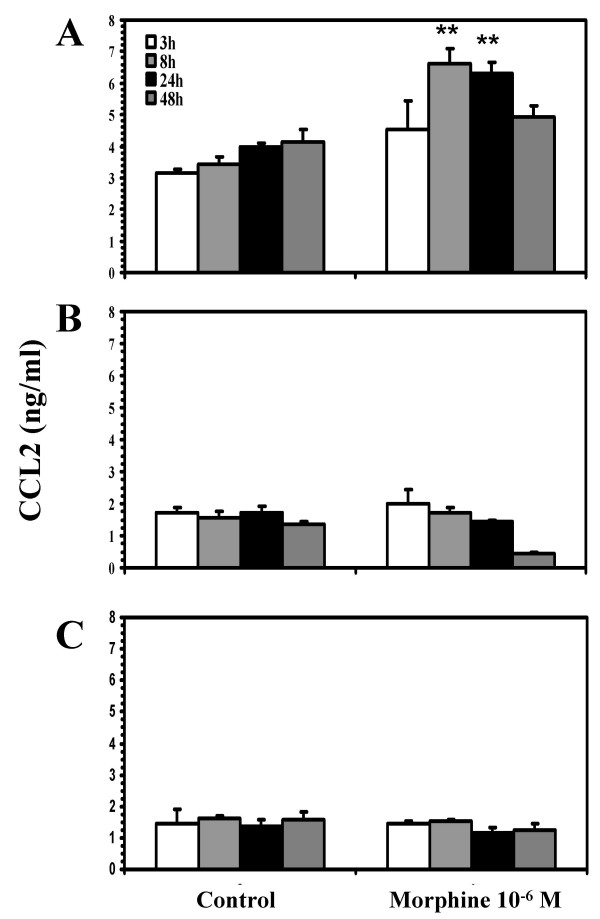
**Effect of morphine on CCL2 production by human neurons, microglial cells, and astrocytes**. Cell culture supernatants were collected from A) highly enriched neuronal cell, B) microglial cell, and C) astrocyte cultures treated with medium (control) or morphine (10^-6 ^M) for the given time points and assayed for CCL2 by ELISA. Data are mean ± SD of triplicates and are representative of three separate experiments using cells derived from different brain specimens. **P < 0.01 versus control.

**Figure 4:**
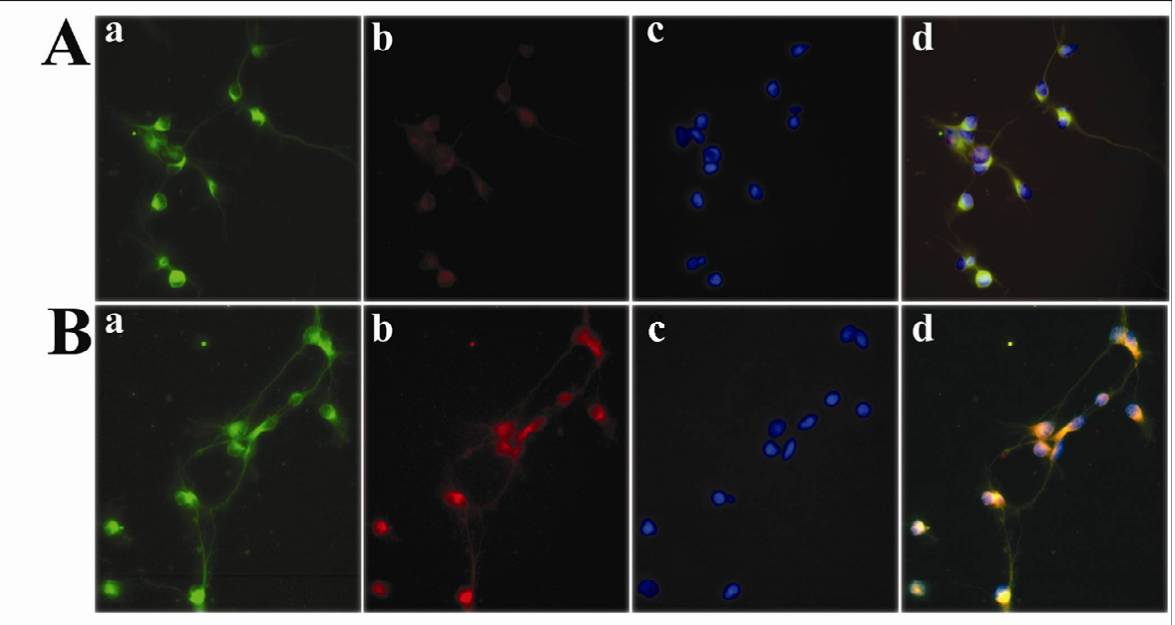
**Morphine effect on CCL2 production by human neurons**. Immunocytochemical staining of highly purified (>95%) human neurons incubated with (A) medium and (B) morphine (10^-4 ^M) for 24 h was performed with (a) MAP2 antibody (neuronal marker, green), (b) CCL2 antibody (red), and (c) nuclear DAPI stain (blue). Colocalization of MAP2 and CCL2 is shown in the merged images (d).

To determine whether morphine stimulates expression of CCL2, highly enriched neuronal cell cultures were treated with morphine (10^-8 ^M or 10^-6 ^M) for 24 h or 48 h followed by total RNA isolation for RPA. As shown in figure 1, of the chemokines studied, only CCL2 mRNA was expressed constitutively and this was the only chemokine mRNA that was significantly upregulated by morphine exposure. Using an ELISA to measure CCL2 protein, the stimulation of CCL2 production by morphine was found to be both concentration-dependent, with maximal effect at 10^-6 ^M morphine (Fig. 2) and time-dependent, with the most robust effect observed at 8 and 24 h (Fig. 3A). To further confirm that neurons were producing CCL2 in response to morphine, immunocytochemical staining was performed on highly purified (>95%) neuronal cultures, which colocalized CCL2 to cells of a neuronal phenotype and further demonstrated that morphine stimulates neuronal CCL2 production (Fig. 4). The viability of the neurons exposed to morphine 10^-4 ^M was confirmed by MTT and apoptotic assays (data not shown).

### Morphine does not enhance CCL2 production in human astrocytes and microglial cells

To further assess if morphine's stimulatory effect on CCL2 production is specific to neurons or more generally affects CCL2 production by glial cells (microglia and astrocytes), we performed experiments to test morphine's effect on CCL2 production in primary cell cultures of human microglial cells and astrocytes. As shown in figure 3B and 3C, while both microglia and astrocytes constitutively expressed CCL2, morphine exclusively enhanced the expression of CCL2 in neurons, but not in microglia and astrocytes.

### Morphine stimulation of CCL2 in human neurons involves MORs

**Figure 5:**
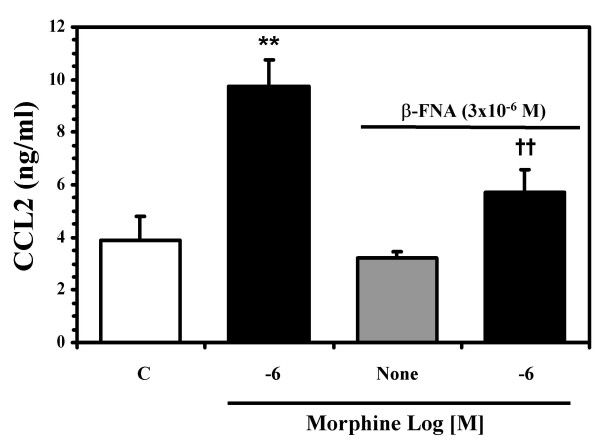
**Effect of MOR antagonist on morphine-mediated stimulation of neuronal CCL2 production**. Highly enriched neuronal cultures were pretreated with β-FNA (3 × 10^-6 ^M) for 30 min prior to morphine (10^-6 ^M) treatment for 24 h. Supernatants were collected for CCL2 ELISA. Data are mean ± SEM of triplicates of three separate experiments using neurons derived from different brain specimens. The saturated two-way ANOVA model fit the data well with a R^2 ^of .91 and Levene's test showed no inequality of the group-wise variances (p = .36). The mean inhibitory effect of β-FNA was estimated to be 3.4 with 95% confidence interval (1.2, 5.6). **P < 0.01 versus control; ††P < 0.01 versus morphine treatment.

Having demonstrated that morphine specifically influences neuronal CCL2 production, we set out to identify if this process is mediated by the MOR. Using the MOR selective antagonist, β-FNA, we demonstrated significant but incomplete blockade of morphine's effect on neuronal CCL2 production (Fig. 5). As morphine also acts at kappa opioid receptor (KOR)s and delta opioid receptor (DOR)s, the partial blockade by β-FNA suggests that morphine-induced stimulation of CCL2 production could be occurring via one or both of these receptors as well as MORs. That KOR involvement seems unlikely was suggested by an experiment using the KOR ligand U50, 488 (10^-6 ^to 10^-12 ^M) which was found to have no stimulatory effect on neuronal CCL2 production.

## Discussion

The purpose of this study was to explore the influence of morphine on CCL2 expression by human neurons. Our focus was on CCL2 primarily because of accumulating evidence of the important role of this chemokine in neuroinflammation and the potential involvement of CCL2 as a communication signal in the cross-talk between the brain and the immune system. In the course of testing the hypothesis that morphine would stimulate CCL2 production by neurons, three observations were made: 1) CCL2, which was the only chemokine examined that was constitutively expressed by neurons under our experimental conditions, was significantly upregulated in neurons by exposure to morphine; 2) morphine's enhancement of CCL2 was specific for neurons, as witnessed by a lack of response of astrocytes and microglia to morphine under our experimental conditions; and 3) morphine's potentiation of neuronal CCL2 production involves the MOR.

Morphine has previously been shown to stimulate CCL2 expression in other cell types [5], and other investigators have shown that neurons constitutively express CCL2 [9-13]. However, this study demonstrated for the first time that morphine can stimulate production of CCL2 by human neurons. Generally, astrocytes and microglia have been regarded as the main brain cell sources of this important chemokine [7], and indeed we demonstrated that both glial cell populations do express CCL2 constitutively. Other investigators have shown that the combination of HIV-1 Tat protein and morphine increased the release of CCL2 from astrocytes [25] and subsequently promoted the chemotaxis of microglia [20]. However, they found as did we, that exposure of astrocytes to morphine alone had no significant effect on CCL2 production [25]. Furthermore, treatment of human astrocytes with morphine has been shown by others to downregulate CCL2 mRNA and protein expression [6].

While the MOR selective antagonist β-FNA significantly abrogated morphine's effect on neuronal CCL2 production, the blockade was only partial. In addition to activating MORs, morphine has the ability to stimulate KORs and DORs. Our observation that the KOR selective agonist U50, 488 did not enhance neuronal CCL2 production, suggested that morphine's effect is not acting through KORs. However, this finding doe not preclude the involvement of DORs or of a non-opioid receptor mechanism in morphine-induced stimulation of CCL2 production. Also, there is the possibility that morphine's stimulatory effect is countered by inhibitory effects of KORs or DORs activation, as such "yin and yang" effects have been commonly seen in previous studies of MOR and KOR agonists in glial cell cultures. Further investigations will be required to tease out whether any of these possibilities are operative.

The biological significance of the findings in this study is unknown, and the results must be interpreted with caution given the artifactual nature of our in vitro culture systems. However, one potential implication of the specificity for neurons of morphine's stimulatory effect on CCL2 production is that opiates may increase recruitment of inflammatory cells within the CNS via their effect on neurons. Such opiate-mediated expression of CCL2 may hypothetically be beneficial, as demonstrated by the observation that CCL2 protects human neurons and astrocytes from NMDA or HIV-tat-induced apoptosis [21], or deleterious, as CCL2 plays a key role in recruiting HIV-infected leukocytes into the CNS [15], and recruitment of inflammatory cells in itself may expose neurons to toxic mediators [26]. Finally, while the focus on CCL2 in this study was based on growing evidence of that CCL2 plays a pivotal role in neuroinflammation, other chemokines that are produced by neurons, such as the CX3C chemokine fractalkine [27], may also be important signals whereby neurons recruit inflammatory cells within the CNS.

## Conclusion

Taken together, the findings in this study support the hypothesis that morphine stimulates CCL2 expression by human neurons and add another mechanism to a growing repertoire whereby opiates could alter the neuroinflammatory process.

## Competing interests

None of the authors has a commercial or other association that might pose a conflict of interest with the current study.

## Authors' contributions

RBR participated in the design of the study and was responsible for writing the manuscript. SH carried out the isolation of neurons and glial cells and the immunoassays. WSS performed the RPA and carried out the statistical analysis. PKP conceived of the study and participated in its design and coordination. All authors read and approved the final manuscript.
